# The Impoverished Publicness of Algorithmic Decision Making

**DOI:** 10.1093/ojls/gqae027

**Published:** 2024-08-10

**Authors:** Neli Frost

**Keywords:** public decision making, artificial intelligence, machine learning, public administration, administrative law, law & technology

## Abstract

The increasing use of machine learning (ML) in public administration requires that we think carefully about the political and legal constraints imposed on public decision making. These developments confront us with the following interrelated questions: can algorithmic public decisions be truly ‘public’? And, to what extent does the use of ML models compromise the ‘publicness’ of such decisions? This article is part of a broader inquiry into the myriad ways in which digital and AI technologies transform the fabric of our democratic existence by mutating the ‘public’. Focusing on the site of public administration, the article develops a conception of publicness that is grounded in a view of public administrations as communities of practice. These communities operate through dialogical, critical and synergetic interactions that allow them to track—as faithfully as possible—the public’s heterogeneous view of its interests, and reify these interests in decision making. Building on this theorisation, the article suggests that the use of ML models in public decision making inevitably generates an impoverished publicness, and thus undermines the potential of public administrations to operate as a locus of democratic construction. The article thus advocates for a reconsideration of the ways in which administrative law problematises and addresses the harms of algorithmic decision making.

## 1. Introduction

The use of algorithmic—including machine learning (ML)—models in public decision making to assist or replace human administrators in their routine decision-making tasks, has garnered much attention in recent years.^1^ Scandals such as the ‘Robodebt Scheme’ in Australia^2^ or the childcare benefits scheme in the Netherlands^3^ offer stark examples of why legal scholars are increasingly concerned by this use. From a legal standpoint, such use may certainly contribute to vital features of a properly functioning public administration, such as efficiency, expediency (together referred to as ‘scalability’^4^) and ‘Weberian instrumental rationality’.^5^ But it also potentially undermines both ethical and legal principles that are decidedly significant in this arena, such as fairness, due process, the rule of law, a range of human rights and principles of justice, as well as individual autonomy or dignity.^6^ Concerns that centre on these principles all meaningfully problematise the use of algorithmic models in public administration. Algorithmic decision making is indeed often biased, is typically opaque and unexplainable, and can result in unjust, rights-infringing decisions at least some of the time.^7^

The present article joins these concerns, but offers a different, complementary frame to problematise the use of algorithmic models—particularly ML—in public administration. Broadly preoccupied with the tensions between novel technologies and *democracy*, this frame centres a unit of analysis that is constitutive of the very idea and fabric of democracy: the ‘public’. The article grapples with the following interrelated questions: can ML-driven public administrative decisions be truly ‘public’? And, to what extent does the use of ML models compromise the publicness of public decision making and decisions? These questions are part of a broader inquiry into the myriad ways in which digital and artificial intelligence technologies transform the very fabric of our democratic existence by mutating the ‘public’.^8^

The main claim I advance in the article is two pronged. First, I offer to view public administrations as important sites of democratic construction insofar as they maintain a quality of publicness. To make this argument, I offer a theory of publicness that is tailored to the arena of public administration, and explain the importance of this attribute for the potential of bureaucracies to function as coherent entities in modern democratic states. Secondly, I argue that the increasing deployment of ML technologies compromises the publicness of administrative decision making and decisions to generate an *impoverished* publicness and thereby destabilise this site’s democratic potential.

Together, these two prongs contribute to thinking in the fields of law & technology, public law theory & democratic jurisprudence and administrative law. To the first, the article cautions that the challenges that it identifies are likely to persist even where technological advancement will allow the overcoming of concerns that relate to the bias and opaqueness of ML models, which currently occupy much of the literature. To the second, it offers a view of publicness that complements parallel treatments of this concept, but that is tailored to the specific site of public administration and its unique features, and is also well suited to address the challenges that bureaucracies face today in the advent of technological developments. To the last, it highlights administrative law’s existing limits in fully addressing the plights of algorithmic decision making, and points to novel sites for regulatory intervention.^9^

The arguments the article puts forward unfold as follows. Section 2 addresses the first prong of the argument. It theorises what publicness means in the context of public administration—as both a norm and an institutional practice. Publicness in this account centres on the web of interactions between public administrators themselves and between them and the public’s elected representatives. It is theorised as an attribute that relates to public administrations’ praxis of decision making and to their decisional outcomes. Briefly, it is grounded in a view of public administrations as ‘communities of practice’^10^ that operate through dialogical, critical and synergetic interactions driven not only by explicit knowledge, but also by tacit, practical forms of knowledge. Publicness is further grounded in the view that this unique feature of the fabric and operations of public administrations potentially allows them to track—as faithfully as possible—the *public’s* intricate view of its interests and to produce decisions that reify those interests.

This theory of publicness is grounded in a theory of democracy that explains its normative value. The normativity of publicness lies in the claim that it imbues public administration—that unelected, democratically suspect stratum of state functionaries—with democratic legitimacy by institutionalising the neo-republican ideal of liberty as non-domination within the bureaucracy.^11^ Publicness is also grounded in a theory of the state that helps frame its ontology. I draw here on the work of Martin Loughlin, and his account of power, politics and representation, to explain the political and institutional constraints in which public administrations operate and which shape their task of governing.^12^ Importantly, my concept of publicness is equally grounded in empirical accounts of how public administrations operate in practice, which demonstrate its plausibility. I draw here on literature in both law and political science that empirically examines the nature of interactions between public administrators themselves and their interactions with the public’s elected representatives.

The article then proceeds in section 3 to address the second limb of the argument that problematises the use of ML models in public administration. I begin here by situating the claim in the broader literature on algorithmic public decision making, and offer an overview of the types of normative concerns that have attracted legal scholars’ attention in this context. I then move to offer a different problematisation that draws on my analysis of publicness and the necessary conditions for its viability. Here, I suggest that the use of ML models in public administration is malignant to the operations of communities of practice. The claim, in brief, is that the knowledge and operational logic of ML models is largely incompatible with the types of knowledge and interactions that drive communities of practice. ML models thus undermine the quality of publicness, so that public decision making that incorporates these models will feature an *impoverished* publicness. On this account, publicness is not only deeply political, but also deeply human. I conclude with the observation that if this is the case, the analysis of publicness should inform how we shape the law that regulates ML systems.

## 2. A Tailored Theory of Publicness

The notion of publicness (much like that of the ‘public’^13^) is a foundational element of democratic governance. It is also, however, multi-dimensional, and links to the ideal of democracy through many nodes which have been the object of much political and legal theorising. Often referred to as ‘publicity’, publicness has been theorised in relation to different sites of democratic construction, as a feature of, for example, political action,^14^ public discourse^15^ and law.^16^ These theorisations have infused the vague concept of publicness with content or meaning that derives from the specific attributes of the site in which it is theorised.^17^ In all of these sites, however, publicness functions to create or bolster democratic legitimacy by ensuring the public’s potential to continuously shape political structures and outcomes.

The article joins these accounts of publicness, but theorises its meaning in yet another potential site of democratic construction, albeit a particularly contentious one. It focuses on public administration, which is a key institutional feature of modern democracies, yet one in which the ideal of democracy as ‘rule by the people’ runs into considerable difficulties. Indeed, the democratic nature of the bureaucracy has been subject to much scrutiny and debate.^18^ Exploring the idea of publicness in *this* context thus aims to supply a democratic justification for the bureaucracy, which merges democratic theory, theories of the state and knowledge about the practical realities of public administrations’ operations. Such a justification is particularly timely in light of the novel challenges that bureaucracies face in the advent of technological developments.

The concept of publicness that I put forward moves beyond references to prominent principles that are usually enlisted in either defence or critique of the democratic nature of the bureaucracy, such as transparency, legality, rationality or proportionality of public decision making and decisions. Rather, it is grounded in a view of public administrations as communities of practice, centring on the types of interactions fostered within these communities and on the types of knowledge they require. It suggests that these communities, their interactions and knowledge cultivate a culture and ethos of decision making that underpin the bureaucracy’s potential to track (as faithfully as possible) the public’s heterogeneous view of its interests, so as to perform the democratic function of producing decisions that reify these interests (to the best extent possible).

This account of publicness, on which I expand below, is underpinned by theories of democracy and a theory of the state. These theories help explain why publicness is important, but also why it is at all plausible—why it is coherent with the framework of the modern democratic, administrative state. Beginning with its importance, a bureaucracy that has the potential to track the public’s heterogeneous view of its interests and to produce decisions that reify them is one that can institutionalise the neo-republican ideal of liberty as non-domination.^19^ While the notion of publicness that I endorse may indeed align with other visions of democracy,^20^ neo-republicanism offers a particularly strong normative grounding for it. Briefly, neo-republican theory offers a conception of democracy that is not principally averse to the idea of an interventionist administrative state. It does, however, grant democratic legitimacy to exercises of power by unelected officials if these are subject to the polity’s influence and control such that decisional outcomes continuously track the *polity’s* view of its interests.^21^ Neo-republicanism, then, perhaps contrary to competing theories of democracy, prioritises the potential of, and sets a framework for, political communities to constitute themselves as *publics*. The notion of publicness that I offer caters precisely to that aim. Public administration that features publicness would thus function as a site of democratic construction, in which (along with other sites) political communities are able to self-constitute as publics.

The view of publicness as grounded in the *modus operandi* of public administrations, their praxis of decision making and in the features of their decisions is also importantly undergirded by a particular view of the very nature, purpose and constraints of public administrations that characterise them as institutions of the state.^22^ This institutional view draws primarily on the work of Martin Loughlin. Loughlin illuminates the irreconcilable tension that characterises the activity of governing, between the promotion of community well-being on the one hand and preserving individual autonomy on the other.^23^ According to Loughlin, it is this inherent discord in the act of governing that gives birth to the practices of *politics*: to those practices rooted in conflict through which people collectively mediate between different, often opposing visions of how they should live.^24^ Loughlin addresses this discord by suggesting that when the practices of politics are institutionalised through structures of authority—as is the case in modern representative democracies—they also generate a ‘power to’: the power of a people to act in concert as a *public*, and to channel political conflict into political ends.^25^ Yet, while this channelling affords public decision making its ‘public’ nature, and gives rise to a distinct form of public agency,^26^ it is still plagued by the absence of unequivocal and commanding benchmarks that effectively guide the fundamental choices that public decision makers need to make between community members’ rival interests and ideals.^27^ Loughlin terms this existential political predicament the ‘brokenness of politics’,^28^ emphasising that the public interest is not monolithic, but rather inherently and irreparably dialectic and contentious.^29^

Loughlin’s analysis thus frames the existential predicament of public administrations that prescribes their unique *modus operandi*, and on which the theory of publicness builds: the predicament of needing to make choices about public policy in the absence of undisputable instructions from the electorate about which choices to make.^30^ In the absence of a monolithic vision of public interests that can simply be identified as such, the task of public decision makers becomes one of tracking the public’s non-coherent, non-static view of its interests, and of producing decisions that can best reify these interests. The complexity of this endeavour designates public decision making as a practical activity that is directed by experience and underpinned by a ‘prudential method’— by reasoning that is guided by rules, but the precise meaning and application of which is necessarily sensitive to, and navigates between, dynamic and changing circumstances.^31^ This prudential method best enables tracking—as faithfully as possible—the public’s intricate view of its interests.

Loughlin’s theory thus frames the practical and institutional constraints within which public administrators operate, and therefore scaffolds the ontology of publicness as an attribute that is defined by reference to the unique features and functions of the bureaucracy. This ontology breeds two important questions: how do the prudential methods of public decision makers look like as a praxis? And how do they generate conditions under which it is possible to best track the public’s complex and heterogeneic view of its interests in order to remain faithful to the public and satisfy the ideal of liberty as non-domination?

I suggest that the answers may lie in the vision of public administrations as communities of practice that feature certain types of knowledge, certain types of interactions and certain types of decision-making practices. The notion of communities of practice originates in the work of Jean Lave and Etienne Wenger, on which Wenger then expanded.^32^ It describes ‘the communities through which individuals develop and share the capacity to create and use knowledge’,^33^ and involves processes in which people actively partake in communities’ social practices and through which they construct ‘identities in relation to these communities’.^34^

The notion of publicness that I offer extends Wenger’s ideas to describe the institutional context and culture in which a dialogical, co-ordinative, synergetic and responsive engagement can take place between public administrators themselves, and between them and the public’s delegates—who together represent the state.^35^ This vision, however, is not purely a theoretical one. It maps onto the material realities of public administrations as communities of experts as these are described in extensive empirical literature in both law and political science. One important contribution to this view is the work of Elizabeth Fisher and Sydney Shapiro on administrative competence and expert administrative capacity.^36^ Public administrations are described in Fisher and Shapiro’s account as communities of experts that operate on the basis of dialogical, relational principles. These enable the co-ordination of a range of skills, knowledge practices and experiences within an institutional context and culture that form the fabric of public administration and that require ongoing cross-talk and exchange of countering views and critiques.^37^ This is a thick view of administrative expertise that moves away from the thin imagination of public administration as either a mechanical, technocratic endeavour or as undercover politics.^38^

On this account, administrative expertise is not an individual expertise, but rather a synergetic, *institutional* expertise that is more than the sum of its parts. It consists not only of formal training and ‘explicit knowledge’, but, more importantly, of ‘tacit knowledge’ that is produced through the social process of constant deliberation, critical reflection and deep institutional immersion.^39^ Originating in the work of Michael Polanyi, the term ‘tacit knowledge’ refers to an embodied form of assimilated knowledge, or ‘know how’.^40^ It is a practical form of knowledge rather than a technical one: it cannot be learned, taught or articulated through structured or mechanical rules, but can only be acquired and applied through action. It is—together with technical knowledge—constitutive of any skill,^41^ and is underpinned by a personal familiarity with nuances and standards of behaviour, which could only be communicated through and obtained by continuous practice and experience.^42^ Polanyi exemplifies this idea in reference to art:

Rules of art can be useful, but they do not determine the *practice* of an art; they are maxims, which can serve as a guide to an art only if they can be integrated into the *practical knowledge* of the art. They cannot replace this knowledge.^43^

Tacit or practical knowledge is thus the type of knowledge that, together with explicit knowledge, underpins the prudential method of decision making.^44^ It is a communal knowledge, involving a profound familiarity with the system’s culture and its ‘informal moral taxonomies’^45^ and ‘conventional wisdoms’,^46^ as well as a capacity to exercise reasoned analysis and contextual judgments that fit within the legislative frameworks by virtue of which the system was made.^47^ The tacit knowledge of public administrators can thus be equated to a form of craftmanship or a local knowledge of a ‘vernacular’ character;^48^ a set of professional skills embedded in practice, and based as much on intuitions, implicit learning, contextual insights, awareness, effective communication, critique and argumentation as they are on structured method and formal training.^49^ These insights, awareness and praxis of critique and argumentation are ‘local’, in the sense that they all have their own structures of meaning embedded in webs of significance that are created by public administrators who partake in them, and in which their administrative expertise partly lies.^50^

This form of administrative expertise is further characterised by Fisher and Shapiro as an inherently co-ordinated one, involving the amalgamation of a multiplicity of expertise types which all involve tacit forms of knowledge, such as ‘interactional expertise’ (the ability to master the language of a domain without, necessarily, the capacity to practice it) or ‘decision-making expertise’ (the ability to reconcile conflicting evidence and perspectives in order to produce decisions).^51^ It also involves the capacity to make ‘appreciative judgments’ that include an openness to search, dynamic and shifting normative evaluations of what is important and an investment of oneself in the situation. Communities of practice, in other words, operate as what Geoffrey Vickers has termed ‘appreciative systems’.^52^

These various modes of expertise are also complemented by a more general institutional and practical expertise in overcoming political or monetary barriers in order to get things done, alongside the necessary skills to explain and justify decisions in ways that cater to their relevant legal constraints and associated normative rationales.^53^ Part of what helps create and sustain these communities of practice are a set of formal and informal institutional norms that are fostered as part of expertise, and that collectively guide public decision makers and ensure a culture of common responsiveness between them, as well as a sense of duty and professionalism.^54^ They are key to establishing the ‘characteristic modes of thought’^55^ that are related to ‘doing’ public administration.^56^ In the US context, for example, such norms are referred to as ‘internal administrative law’.^57^ They are generated by agencies themselves to govern and supervise agency personnel as well as interagency engagement, and to generally guide the internal interactions between public administrators and different agencies.^58^ They help guarantee that public decision makers are able to execute their authority by reference to how this authority is executed by other officials.^59^

Publicness thus lies in the ways in which these expertise types and the ongoing dialogical, critical engagements that they both foster and demand cultivate the potential to best track the public’s heterogeneic view of its interests in a given context. They do so, first, by creating the conditions under which agencies might be precluded—to the extent possible—from acting on any one administrator’s personal view of what public interests demand in any particular case. But most importantly perhaps, these knowledge practices and interactive procedures create an ethos of public decision making that allows agencies to continuously be sensitive to, and process, multiple, diverse and at times conflicting inputs from the public. They then allow public administrators to employ prudential considerations and operate successfully within the inescapable reality of dynamic and shifting politics: the reality of malleability, of conflicting and irreconcilable normative aims and of the ever-present inherent tensions between differing interests and perspectives.^60^ From the point of view of publicness, it is this ethos or culture of decision making that matters, insofar as it nurtures a particular environment within which specific decisions—ranging from bigger-stakes decisions to run-of-the-mill decisions—are then made, and within which the *modus operandi* of public decision making as an idiosyncratic practice is formed.^61^

There is, however, another crucial component to publicness that produces this potential. The capacity to track the public’s complex and dynamic view of its interests, and produce decisions that best reify them, depends not only on the expertise of and dynamics between public administrators themselves, as described above, but also, importantly, on the ongoing engagement between public administrators and their elected *political* counterparts, who represent and interact with the public in a democracy^62^—or, as framed by Katherine Strandburg, on the engagement between ‘agenda setters’ and ‘rulemakers’.^63^ This second dimension of publicness thus demands that we expand the notion of the community of practice to understand it as a space that structures integrated interactions between the administration and the legislator;^64^ a space in which the operations of public administrators could, on the one hand, be supervised by, guided and re-evaluated by elected political offices, and in which public administrations could influence agendas bottom-up, on the other.^65^

As the discussion of Loughlin’s theory has made clear, the production of decisions that best embody public interests is almost by definition an imperfect endeavour, given the existential conditions of democracies: the fictitious nature of a collective consciousness, the brokenness of politics and the need for representation.^66^ These existential constraints mean that decisions that reify public interests are never simply identified, but rather are produced: they emerge from interactions that generate the best conditions for their production. From this perspective, publicness requires that communities of practice involve more than horizontal deliberative exchanges, shared points of reference, co-ordinated interactions and mutual critiques amongst public administrators themselves; but also *vertical exchanges* between public administrators and the public’s elected representatives that are baked into the everyday life of public administrations and that are an essential aspect of generating public agency.^67^ These vertical interactions allow for additional sensory inputs from the public that are filtered, here, through its representation in the legislator. Such mechanisms clearly exceed the formal legislative mandates that give rise to and scaffold administrative authority. They also vary considerably between jurisdictions and particularly between parliamentary and presidential systems. While a comprehensive account of these mechanisms is beyond the scope of this article, a few examples are instructive.

In the US, for instance, research in political science highlights the ‘dual dynamics’ that characterise interactions between the bureaucracy and both Congress and the President.^68^ These dynamics are a product of top-down, bottom-up, *ex ante* and *ex post* interactions, which include, among others: oversight systems such as hearings, investigations, information and reporting requirements;^69^ budgetary constraints and reviews;^70^ or reward and sanctions of public administrators. They also include administrative procedures that generate a bureaucratic ecosystem that reflects well the political dynamics that generated policies further upstream.^71^ These latter procedures include the set of formal and informal internal administrative laws that allow the translation of political priorities into agency action, but also agency knowledge into political priorities.^72^

Some of these procedural requirements—such as the notice and comment rules in the Administrative Procedure Act, for example^73^—are realised in the relationship between public decision makers and affected stakeholders, but also function to ensure political direction by Congress and the President. They do so by generating ample opportunities for agenda setters to respond—even if cautiously^74^—to agencies’ attempts to further unwelcome decisions.^75^ Another mechanism for top-down political oversight is the authorisation of advisory committees by Congress to direct administrative agencies in their decision-making processes.^76^ Although advisory committees may vary considerably in their precise operations, their overall purpose is to guarantee that regulatory debates are informed by the same perspectives that structure and frame legislative debates.^77^ Mechanisms of a similar nature exist in parliamentary systems. These include tools of ‘statutory control’—the writing of detailed legislation in order to preclude agencies from acting contrary to the interests of the legislator,^78^ as well as legislative review of executive policy making.^79^

But an equally important aspect of this dual dynamic involves the bottom-up processes that begin with information reporting by bureaucracies and their formulation of problems that then feed into the steering prioritisation of this information by legislators in the course of agenda setting. On this view, part of the role of public administration—and part of the reason it requires a degree of autonomy—is to detect and monitor policy problems and to generate information about these problems that can be transmitted to elected officials for their consideration.^80^ A key feature of the interactions between the bureaucracy and elected representatives is thus that of feedback, through which administrative agencies are also able to drive and shape legislative processes.^81^ The authority delegated by legislators to public administrations is therefore not simply implemented by the latter. Public administrations detect and define problems within their respective spheres of operation, they generate information about these problems and this information is employed by elected politicians in policy making, which then reorients public administrations.^82^

It is, then, the nature of dialectic, bidirectional, feedback-based interactions between the public’s political representatives and expert public administrators within communities of practice that underpin the praxis of prudential decision making and its quality of publicness. It is these practices, and the knowledge that informs them, that maximise the sensory input that allows a tracking of the public’s complex, dynamic view of its interests, but also its filtering through expertise in order to produce decisions that reify these interests. These very practices thus also hold the potential to mitigate the majoritarian influences that these sensory inputs may have on the production of public decisions. The aforementioned examples highlight that publicness is thus found not only in the ethos of decision making that holds back *administrations* from acting on their own conception of what public interests are, but also in the potential of this expertise-based ethos to mitigate the undue impact of often undemocratic *legislatures* captured by majoritarian politics, populism or self-interested politicians.^83^

This account of publicness is thus one that seeks to amalgamate two diametrically opposed approaches to the legislator–administrator relationship: a ‘progressivist’ approach that calls to maximise an apolitical, rational-legal bureaucracy precisely because of the worry about adverse political influence and a ‘neo-democratic’ account concerned with bureaucratic accountability.^84^ It is the multi-dimensional nature of both interagency and legislative–administrative interactions that ensure that agency expertise, interagency dynamics and street-level knowledge counteract the undemocratic tendencies of corrupted legislators much in the same way that legislative oversight and direction counteract captured or biased administrations. Under these conditions, not only is political ‘power to’ and public agency generated, but also decisions that best reify the public’s view of its interests can emerge.

This, of course, may not always be the case, and I do not purport to theorise away the existence of many forces that may and do interfere with this potential. But it is, nevertheless, a structural potential that has material and institutional viability. The theory of publicness, then, although drawing on normative propositions, is a non-ideal theory that draws as much on plausible assumptions about politics as it does on empirical accounts of the operations of public administrations. These empirical accounts demonstrate that public administrations often *do* operate as communities of practice. And when they do, the unique ethos and culture of these communities offer substantial structural advantages that plausibly allow bureaucracies (even if not always) to make good on their democratising potential to reify public interests within the constraints imposed by the brokenness of politics. Public administrations, on this view, can function to institutionalise the neo-republican ideal of liberty as non-domination and thus operate as another locus for democratic construction in which political communities continuously constitute themselves as publics.

## 3. Algorithmic Decision Making and an Impoverished Publicness

The main argument I make in this section is that the use of algorithmic—particularly ML—models in public decision making alters the composition of communities of practices in ways that fundamentally disrupt their operations and interfere with the potential of these communities to track the public’s view of its interests and reify these interests via their decision-making processes. This is a conceptual claim, which I illustrate through some examples. In disrupting the ontology of these communities, algorithmic decision making thus undermines some of the formative features of complex democratic societies built on multiple dialogical spheres of engagement, interaction and critique. The conclusion I draw here is that there is something deeply ‘public’, and therefore deeply and inherently human, about these communities.

The important point I wish to emphasise in this analysis, however, is not that communities of practice always function in optimal ways. As previously mentioned, there are ample discussions of personal or institutional biases and private interest groups that capture public administrations^85^ and of undemocratic trends that plague legislators, which together prevent communities of practice from functioning in the ways that I describe. Nor is my claim that ML models introduce challenges that we have never encountered before. Indeed, much of the literature on which my notion of publicness draws is troubled by certain accounts of *human* decision making that are incompatible with the features that I suggest characterise administrative communities of practice. Further, much of this literature long predates the existence of the technologies at the crux of this article.^86^ My claim, rather, is that, irrespective of these challenges, the use of algorithmic—particularly ML—models undermines the *potential that does exist* for public administrations to operate in ways that preserve publicness. I argue that this is because of features that are inherent to the knowledge and operational logic of these technologies. The proposition that administrative communities of practice are, by nature, deeply human does not therefore entail that when public administrations *are* human they necessarily function as communities of practice.

### A. The Challenges of Algorithmic Decision Making

The problematisation that I offer can be contextualised within the broader literature that examines the challenges raised by algorithmic models to what may be termed democracy’s ‘judicial expressions’: those legal principles that give effect to, and institutionalise, the political ideal of democracy in our daily lives. As a phenomenon, algorithmic public decision making is neither novel nor homogeneous.^87^ For our purposes here, I briefly describe what legal scholars typically talk about when they concern themselves with this phenomenon. Broadly speaking, algorithmic public decision making refers to the use of computational (often ML) models by the judiciary and by administrative agencies to complement or replace parts of those decision-making processes that constitute the everyday lives of these institutions and that were previously handled manually.^88^ The subject of inquiry, therefore, is not only the computational model or mathematical construct itself, but rather the ‘sociotechnical assemblage’ within which the model is embedded.^89^

To offer one well-known example, the COMPAS risk-assessment tool is an algorithmic model whose purpose is to statistically predict the likelihood that a criminal defendant will reoffend on the basis of a set of data points, including those relating to the defendant’s personal history (eg parents’ relationship status) or criminal history.^90^ The model is meant to assist the judge in deciding on a defendant’s pretrial release or parole, by offering a mathematical assessment of the defendant’s probability of recidivism to complement or substitute the judge’s clinical evaluation.^91^ Other examples from the field of public administration (as opposed to the judiciary) include the use of algorithms to predict crime,^92^ tax fraud,^93^ welfare eligibility^94^ or child-safeguarding concerns.^95^ In all these cases, computational models are employed by public administrators to assist their routine decision-making tasks, such as where to send police forces, whose application to flag as suspect or who to investigate for potential child abuse, to name but a few.^96^

These examples direct us to important features of the technologies at focus in this article. While the term ‘algorithm’ may refer generally to any programmed process that transforms input data into some required output,^97^ specifically of interest here are models used for *predictive* purposes, and that do not require specifying in advance what the functional relationships are between variables in the model.^98^ These models mine large (pre- or unlabelled) data sets to autonomously identify correlations and patterns between variables within the data through iterative processes in which the model finds (or ‘learns’) what are the ‘optimal values for the matrix of weights’.^99^ Put within the socio-technical context of public decision making, the model thus assumes an autonomous evaluative role: it offers a way forward by providing a mathematical assessment, given its objective function.^100^

The perceived challenges raised by the deployment of these tools have been subject to a breadth of critical scholarship from within and outside the legal field. One of the earliest responses originated from an intellectual community named FAT/ML (now FaccT).^101^ As its name indicates, the FAT/ML community directed (and continues to direct) its intellectual force to conceptualising and addressing the ways in which the use of ML models impacts the fairness (F) and transparency (T) of public decisions, and to how such decision-making systems could therefore be made accountable (A). This community’s focus on fairness has mainly emphasised bias and discrimination, which are further compounded by algorithms’ inherent opaqueness.^102^ The principal problem of algorithmic decision making is consequently framed by FAT/ML as potentially producing opaque, biased or discriminatory outcomes.^103^

Legal scholars sought to reframe FAT/ML-driven problematisations and solutions by employing legal vocabularies and doctrines, for example, those of human, fundamental or civil rights.^104^ Such bids have been driven by a desire to conceptualise the adverse consequences of algorithmic public decision making as violations of specific, enumerated rights in order to overcome the ostensible vagueness of ‘ethics’ and ‘fairness’ that are taken to lead all too often to ‘ethics washing’^105^ by industry.^106^ Legal frameworks, by contrast, claim to have the advantage of anchoring discussions within well-established and authoritative legal machineries capable of determining concrete harms (such as to the right to privacy) and imposing specific legal obligations on specific actors (such as governments).^107^ Other legal approaches have centred on principles embodied in doctrines of constitutional and administrative law, such as due process or the rule of law.^108^ Yet others have focused on questions of justice, dignity, autonomy, agency or subjectivity.^109^

The analysis that follows joins these vital problematisations, but shifts focus away from the ways in which algorithmic public decision making undermines the ‘judicial expressions’ of democracy to the ways in which it undermines the ‘public’ as a constitutive element of the *very fabric* of democracy. In taking this view, my aim is to shift emphasis from the role of public institutions in securing the rights, interests and status of *individuals* to their role in preserving ‘supraindividual legal interests’^110^—in continuously constituting and reconstituting the public and its interests. This focus on publicness not only targets the unique ‘public’ character of public decision making, but also some of the unique features of *human* decision makers. It illuminates the transformative effects that ML models have on democracy, and points to implications that might not be meaningfully addressed through extant regulatory tools.^111^

### B. The Challenges of ML from the Perspective of Publicness

#### (i) The (algorithmic) community of practice

The operation of ML is almost antithetical to the operations of a community of practice as I described them in section 2, and to the act of governing as it is understood from the perspective of publicness. ML technologies are typically employed to ‘discover, classify, rank, cluster, recommend, label or predict’^112^ something; a process during which ‘The algorithms yield correlations between new data and patterns identified in the data flows to yield a particular, highly data-intensive form of knowledge’.^113^ This mathematical, data-intensive form of knowledge is predicated on the ‘assumption’ that phenomena in the world can be strictly classified into steady and distinct categories.^114^ Algorithmic prediction depends on maintaining these distinctions, and on how these distinct categories or variables are combined or correlated to produce a predictive outcome.

This type of data-intensive knowledge is, however, very different from the type of situated, tacit, collective, experiential knowledge that undergirds administrative expertise within the community of practice; and one that stands in tension with the very activity of governing as a collective ‘science of muddling through’.^115^ From the perspective of publicness, the activity of governing itself is about giving effect to the ‘power to’^116^ by constituting and reconstituting the public throughout time, and generating and regenerating a system of values that attaches to the public.^117^ It is about the continuous exercise of a prudential method of decision making that is highly aware of, and attuned to, the core tension between the preservation of liberty and the furthering of common goods.^118^ It is, in other words, about making unceasing intersubjective ‘appreciative judgments’^119^ that all require both explicit *and* tacit knowledge, the latter of which can only be acquired through collective experience and interaction if it is to have the potential to faithfully track the public’s composite view of its interests.

The knowledge that feeds into, and is produced by, ML models is almost diametrically opposed to the practical knowledge and connoisseurship that characterises communities of practice and is an intrinsic element of their *modus operandi*. It is an extreme form of technical knowledge formulated in strict rules, or code. Instead of emerging through the collective interactions within the community of practice and through co-ordinated, argumentative, discursive-analytical and synergetic exchanges that create spaces for malleable ‘everyday casuistry’,^120^ for contextual insights and for heuristic intuitions, algorithmic decision making is manifestly singular, opaque, structured and methodical. Its decision-making process involves ‘learning’ that is entirely schematic, and is never socially experienced. It involves the mathematical estimation of parameters or the identification of current structures in the data, in the absence of any mutuality, critical reflection or irreconcilable tensions. This frustrates the collective process of valuation in which the intentions and activities of others and informal institutional norms are taken as a guide to conduct.^121^

In other words, ML models are not communal thinkers by design. The type of predictive knowledge they produce may represent well the current properties of the data, and thus perhaps constitutes the ultimate explicit knowledge, which is indeed relevant and important for public decision making. But it does not allow for the flexible contextualisation of this data in ways that can account for the brokenness of politics—for the realisation that people, and their interests, almost never lend themselves to categorical distinctions, and that there is therefore never one given and predetermined answer to policy decisions. Such decisions are always a product of channelling ever-persistent tensions between conflicting interests.^122^

From the perspective of publicness, then, the opaqueness and associated difficulties in the interpretability of ML models thus matter as much for public decision makers *themselves* as they do for those affected by their decisions. From this perspective, what matters is that these models do not draw on, or easily lend themselves to, the critique and inquiry by others within the community of practice which are needed in order to generate the type of contextual intersubjective insights or implicit learning that ensure the development of a *collective* point of view that guides decision making. These features, together with the very knowledge logic of ML, preclude the development of characteristic modes of thought, conventional wisdoms, responsiveness and adaptation that allow public administrators within the community of practice to operate on the basis of mutually developed prudential considerations. ML models are simply driven by a *different* type of expertise. They ‘do’ public administration in ways that are markedly different from those of the community of practice.

To illustrate, in 2013, Dutch authorities introduced an algorithmic system to detect potentially fraudulent child benefit applications. The model, which included ‘self-learning elements’,^123^ was designed to generate applicants’ risk profiles. Applicants flagged as risky immediately had their benefit payments suspended, and were subjected to further investigation by public administrators.^124^ Conversely, applications that were given low risk scores were automatically approved.^125^ From a technical standpoint, the self-learning elements of the model meant that it was able to ‘independently and autonomously learn from experiences over times, and to make changes to how they work without these changes being explicitly programmed by humans’.^126^ So, the more the system learned, the more autonomously the model could ‘decide’ how different risk factors interact to produce a risk score. Of course, the model was not detached from the broader community of practice. It was, indeed, part and parcel of broader decision-making processes in which human decision makers remained highly involved, for example, manually reviewing applications that received the highest risk scores. But those public administrators who reviewed them were never told why the model had produced the risk score that it did.^127^

This childcare benefit scheme generated considerable backlash. Most attention, however, was directed to the system’s discriminatory outcomes. As nationality was one of the risk factors considered by the model, applicants of non-Dutch nationalities received higher risk scores.^128^ There are, of course, other concerns raised by the use of these models, the least of which might be that the model used was simply not a good one. But from the perspective of publicness, an equally concerning issue is that there was nothing ‘communal’ about the ways in which these models reached their outputs. The risk score provided by the model (which is highly consequential for individuals’ lives) was not produced through any dialogical process that included cross-talk and critiques, contextual insights, argumentation or appreciative considerations. Given the very nature of ML models, then, even when they *are* employed as part and parcel of a broader human-led decision-making process, they are never fully embedded within this community in ways that cater to the production of collective articulations of what the ‘right’ course of action might be. Their inherent detachment does not allow human decision makers to secure the decision-making process (to the best extent possible) against any one public decision maker’s view of what public interests require, of becoming determinative of outcomes. The result was that applications were ‘evaluated’ by the model on the basis of the presence of risk factors in the data and the patterns they produced, in the absence of any procedure that would account for the simple fact that there is no one clear answer to the question of which applicants should be flagged and why.^129^ A key feature of public decision making is the uncertainty between means and ends and the ambiguity around what those ends actually are.^130^ The answer to these questions are inherently and deeply ‘public’.

Importantly, the Dutch example also demonstrates how the very inclusion of these models within communities of practice may have the performative effect of slowly degenerating the expertise of each individual administrator within this community, which will then percolate to the communal level. This clearly is a possibility when humans are asked to review high-risk applications without any knowledge of why it is these applications that were flagged. The concern, then, that ML models are malignant for the operations of communities of practice and their potential to centre the tensions inherent in the act of governing may, indeed, not be entirely novel (this critique perhaps resembles other critiques of human decision making).^131^ But these models *do* also introduce challenges that are somewhat distinct from those that came before them: these models obscure the very existence of this tension and thus incrementally reduce democracies’ institutional attention to it. Hence, the more occupational space ML models assume within a community of practice, the easier it is to imagine how they eventually lead to the deskilling of administrators,^132^ as well as to the erosion of the inner processes of contemplation and debate within each individual constituting the community of practice. Consequently, they will also lead to the erosion of that individual sense of obligation, mission orientation, public service and professionalism that characterises administrative expertise and is vital for publicness. Such erosion at the level of individual administrators would then bear substantially on the community of practice as a whole insofar as this community depends on the vigour of interactions between its individual components to generate something that is more than the sum of its parts. In other words, the less generative the contribution and output of each expert within this community, the smaller the contribution made to the cumulative ‘canons’—to that ‘supply of publicly valorized considerations’^133^—that drive the operations and outputs of the community of practice as a whole. In this manner, the effects of ML-driven decisions reverberate beyond the concrete scope of their use and application in specific cases. They gradually mutate the ‘public’.

#### (ii) A (Flawed) Reification of Public Interests

The Dutch case demonstrates another crucial point that goes to the heart of publicness. ML models not only impact the composition and operations of communities of practice, but their use also compromises the reification of public interests. It results in the production of decisions that are not based on criteria that are moulded and fitted through these communities’ ongoing interactions. That is, ML’s decision criteria are not formulated through the dual dynamic between public administrators and the public’s elected representatives that allows for a mutual determination and articulation of what the public view of public interests might be. Decisional outcomes driven by ML are rather determined by the structure of the data, and depend on how the model is set to discover it.^134^ The very categories or variables on the basis of which pattern detection occurs and outputs are produced are ‘decided’ by the model itself, which operates independently of the ‘appreciative system’ that integrative communities of practice constitute.^135^ The use of these models therefore impacts the potential of public administrations to speak in the name of the public, and to produce decisions that reify—to the best extent possible—public interests.

Indeed, the more autonomous ML decision-making processes become, the more ‘evaluative’ and normatively weighty their outputs become. The more autonomous they are, the less knowledge public administrators—let alone agenda setters—have of, and the less control they can potentially exert over, those decision criteria, or distinctions of normative relevance,^136^ that may be determinative of outputs. When it comes to deep-learning models, for example, even those who design them have limited capacity to interpret and explain how precisely the model arrives at a given output.^137^ Hence, when ML models are applied, the processes through which the mutual determination and articulation of what the public view of what public interests demand in specific cases are thwarted. Within this state of affairs, decisional outputs are no longer communally articulated as an outcome of ongoing interactions and tensions between administrators and exchanges with agenda setters. They are made, rather, on the basis of the model’s monolithic ‘view’ of public interests. It is the model that ‘[makes] decisions about value, relevance, and visibility, establishing and allocating privilege’.^138^ The point I want to make here is not one about the (in)capacity of ML models to morally reason in the same way that humans do (although obviously this is not unrelated);^139^ rather, it is that the reification of public interests cannot, by definition, be an individualistic endeavour, but only a public one.

Another example is instructive to demonstrate this point. In 2016, the Allegheny County Office of Children, Youth and Families (CYF) in Pennsylvania introduced a screening tool to mine data to predict which children might—in the future—be subject to abuse or neglect.^140^ Specifically, the model is ‘designed to predict the risk that a child will be placed in foster care in the two years after they are investigated’.^141^ It produces a risk score by weighing various indicators that are proxies for child maltreatment. When the risk score is high enough, the system automatically triggers an investigation which may ultimately result in family separation.^142^ Like in the Dutch example, critiques were launched at the discriminatory outputs and effects of the model.^143^ But again, from the perspective of publicness, there is another, equally concerning issue: that the judgment about what constitutes abuse or neglect is ultimately expropriated from the hands of the public by the model. It is not otherwise made on the basis of the public’s normative, and perhaps contentious views on these very complex and fine-lined questions; but, rather, on the basis of certain proxies identified in the data by the model that produce a monolithic vision of what it is that should be decided.

This is still the case even when one adopts a socio-technical view of algorithmic public decision making which takes into account the fact that ML predictions are merely part and parcel of a more complex decision-making procedure in which human decision makers remain highly involved (both in the design of the model and in deploying it), and in which agency is therefore distributed between the human and the machine. Indeed, in the example just provided, the model was deeply embedded in a broader decision-making process led by humans.^144^ Yet, insofar as algorithmic predictions inform and feed into, or even structure, later stages in the process to generate ‘actionable insights’ in the course of administrative policy making and enforcement,^145^ then the process as a whole is still, to a greater or lesser extent, driven by the model’s ‘own reasoning’ and its ‘own interpretation’ of what public interests require in particular cases, in ways that remain substantially detached from the public and its complex collective consciousness. As one child welfare researcher pointed out:

The very front end of child protection decision-making is understandably the most impactful decisions that you can make in a child’s life, because once you come into contact with the hotline, with an investigator, then your chance of being removed [from home], of course, is increased.^146^

From this viewpoint, ML is indeed not the only decision-making ‘technique’ that detaches decisions from the public. Privatisation, for example, arguably does the same.^147^ But it *is* the case that algorithmic decision making is another, perhaps particularly worrisome, instance of publicness-defying decision making. This is because the opacity of algorithmic systems—the ‘inscrutability of a machine learning model’s computational mapping of input features to outcome variables’^148^—prevents the ‘actionable insights’ they create from being meaningfully embedded within a community of practice. So, while on the one hand such models become entangled in a complex human–computational assemblage, the specific mode of their operation resists a full embeddedness of the type imagined in, and prescribed by the notion of a community of practice that allows the potential reification of public interests.

#### (iii) An Impoverished Publicness

This account of how ML models operate is perhaps not in and of itself a novel insight for those concerned with the intersection between law and technology. What is novel, however, is the conceptualisation of the harms of algorithmic decision making as implicating the publicness of public decision making and decisions: as the harm of compromising the integrative form of communities of practice and their *modus operandi*, and thus their potential to reify public interests. To employ the words of Margaret Jane Radin, ‘conceptualization makes it possible for us to see harm as harm’.^149^

I return now, briefly, to the tailored theory of publicness in order to conceptualise this harm through the idiom of publicness. As the analyses thus far have sought to make clear, the unique operations of administrative communities of practice and the integrative form they take with elected representatives of the public together allow the potential to track—imperfectly, yet as faithfully as possible—the public’s heterogeneous view of its interests, and to produce decisions that reify those interests so as to try and preserve the publicness of public decisions and therefore the democratic legitimacy of the administrative state. Conceptualised as such, publicness functions to maintain the distinction between the personality of private administrators and the decision-making authority that attaches to their post, and to allow political communities to continuously self-constitute themselves as publics. It is thus an important ideal that helps address the irreconcilable tensions that the activity of governing gives rise to, between the pastoral and autonomy-enhancing roles of the state.^150^ In doing so, publicness gives effect to, and institutionalises, the neo-republican ideal of liberty as non-domination in unelected bureaucracies so that these can function as sites of democratic construction.

Clearly, the schematic analyses of the operations of communities of practice do not purport to track the precise operations of public administrations. Obviously, the picture is much more complex, and also involves jurisdictional differences between public administrations, or differences between specific administrative agencies.^151^ Nor were these analyses, as I mentioned already, meant to idealise in any way the operations of public administrations. Rather, the argument is that there is something unique about the operations of public administrations, given how ‘undemocratic’ but fundamentally political (in Loughlin’s sense of the term) they are;^152^ and that perhaps the best way to ensure their potential to operate as sites of democratic construction is by preserving their *modus operandi* as integrative communities of practice that feature publicness.

Publicness in this context is thus more about normativity and less about legality. It is a requirement for the type of expertise-based, interactional, dialogical, co-ordinated, collective, dynamic, mission-oriented, professional and ultimately prudential culture and method of decision making that can only be co-produced within a community practice that integrates public administrators with elected political actors acting on behalf, and in the name, of the public; and through which public agency comes about, and decisions that can best track the public’s intricate view of its interests are made.

All of this is deeply frustrated by the use of ML models in public administration. Public decision making that includes algorithmic—particularly ML—predictions can therefore be conceptualised as featuring an *impoverished* publicness insofar as their processes, and the decisions which they produce, minimally feature publicness as a quality. If so, these models put at risk the potential for public decisions to be made in the name of the polity and in its interests, even if that potential was not always fully realised prior to their introduction. Importantly, the more pervasive algorithmic public decision making becomes, the less capacity will democracies have to notice and *critique* the loss of this potential. The increasing introduction of ML into public administrations will therefore slowly chip away at the very fabric of democracy by mutating the ‘public’.

An impoverished publicness, or the (at least) partial destabilising of the ontology of public administrative decision making, thus links closely to questions about legitimacy and accountability that often arise in the broad literature concerned with the privatisation of government functions,^153^ and, indeed, in the specific context of algorithmic public decision making.^154^ But, as the normativity of publicness suggests, the potential of algorithmic public decision making to result in an impoverished publicness would be most concerning because of how it robs political communities of the continuous and routine political power to constitute themselves as publics, by collectively articulating and determining the conditions under which the lives of their members are governed.

The incorporation of ML in public decision making is therefore disturbing not only because it limits decisions’ explainability and contestability, but primarily because it is inherently limited in its capacity to answer to the requirements of publicness in ways that are bound almost by definition to result in domination. And so, while it may well be that the use of these technologies is attractive for reasons that have very much to do with the proper functioning of public administrations, this use would also require that we accept—as a matter of principle—that some areas of decision making will be dominating insofar as it is the model’s view of what public interests demand that will drive decision making. From this perspective, the normal mechanisms neo-republicanism offers in order to prevent domination might prove ill-fitted to address the impoverished publicness induced by algorithmic decision making.^155^ This is because domination that results from impoverished publicness is not consequential but deontological—it is inherent to decisions led by ML models insofar as these do not allow the public to press its heterogeneous and complex direction on the state. If this is the case, there might be a fundamental difference between being affected by a deficient algorithmic decision maker and a deficient human one. Publicness, it turns out, is not only deeply political, but also—and perhaps consequently—deeply *human*.

## 4. Conclusion

Broadly concerned with tensions between novel technologies and democracy, this article has confronted the question of whether ML-driven public decision making can be truly ‘public’. To do so, the article has first engaged in an inquiry into those unique features of public administrations that both frame and constrain their operations as key institutions within the democratic apparatus. I have argued that, despite the many concerns around the democratic illegitimacy of bureaucracies, public administrations can, and often do, function as sites of democratic construction insofar as they feature a quality of publicness. Publicness, in this context, is theorised as an attribute that relates to the unique *modus operandi* of public administrations as communities of practice.

ML models, however, operate in ways that are malignant for the types of knowledge and interactions that characterise communities of practice and that allow them to faithfully track the public’s heterogeneous view of its interests and reify them. The use of these models in public decision making thus generates an impoverished publicness and undermines bureaucracies’ potential to function as sites of democratic construction through which political communities can continuously constitute themselves as publics. If so, the use of ML technologies adversely affects something more than bureaucracies’ capacity to respect and promote the rights and interests of individuals. It also transforms the ‘public’.

The problematisation that I have offered, then, also bears consequences for how we think about the effectiveness of existing regulatory interventions that law imposes to mitigate the harms of algorithmic decision making. A discussion on regulatory tools has been beyond this article’s scope, but it is worth highlighting, by way of conclusion, the tensions that the notion of an impoverished publicness creates for law, particularly for administrative law.^156^ In the UK, for example, algorithmic decision making is subject ‘to the same doctrinal tools of judicial review as are decisions made by more traditional means’.^157^ But not only does classic judicial review prove problematic as a regulatory tool to address algorithmic public decision making,^158^ its focus on the *individual* decision maker rather than on the community of practice also renders it fundamentally ill-fitted to address the impoverished publicness of algorithmic decisions. The lens of publicness, then, if taken seriously, prescribes a shift of focus for administrative law in the face of technological advancement.^159^ It requires that law pay closer attention to the unique demands made on public administrations to operate in ways that sustain the type of expertise-based, co-ordinated, collective, prudential culture and ethos of decision making that effectively lend public administrations their potential for deep democratic legitimacy.

## Footnotes

1 Such uses are prevalent in eg predictive policing, criminal justice, social welfare and immigration. See generally Cary Coglianese and Lavi M Ben-Dor, ‘AI in Adjudication and Administration’ (2021) 86 Brook L Rev 791; Kiran Stacey, ‘UK Officials Use AI to Decide on Issues from Benefits to Marriage Licences’ *The Guardian* (23 October 2023) <www.theguardian.com/technology/2023/oct/23/uk-officials-use-ai-to-decide-on-issues-from-benefits-to-marriage-licences> accessed 5 July 2024. In this article, I focus on administrative rather than judicial decision making, and limit my focus to public administration within the state, setting aside public decision making with transboundary implications and public administration at the global level.

2 The name ‘Robodebt’ was coined in reference to the Australian government’s automated welfare debt calculation and collection programme. The Royal Commission into the Robodebt Scheme, Report (7 July 2023) <https://robodebt.royalcommission.gov.au/system/files/2023-09/rrc-accessible-full-report.PDF> accessed 5 July 2024; Monika Zalnieriute, Lyria Bennett Moses and George Williams, ‘The Rule of Law and Automation of Government Decision-Making’ (2019) 82 MLR 425.

3 Amnesty International, ‘Xenophobic Machines: Discrimination through Unregulated Use of Algorithms in the Dutch Childcare Benefits Scandal’ (2021) <www.amnesty.org/en/documents/eur35/4686/2021/en> accessed 5 July 2024.

4 Nicolò Pagan and others, ‘A Classification of Feedback Loops and Their Relation to Biases in Automated Decision-Making Systems’ arXiv: 2305.06055v1 [cs.CY] (10 May 2023) 1.

5 Katrina Geddes, ‘Computational Prediction: The Co-Evolution of Law and Technology’ (JSD dissertation, NYU School of Law, May 2023) (manuscript on file with author) 55, fn 353, quoting Blake Emerson, ‘Administrative Answers to “Major Questions”: On the Democratic Authority of Agency Statutory Interpretation’ (2016) Yale Law School Student Scholarship Paper 131, 29.

6 See, eg, Reuben Binns, ‘Human Judgment in Algorithmic Loops: Individual Justice and Automated Decision-Making’ (2022) 16 Regulation & Governance 197, 201; On racial justice, see eg Aziz Z Huq, ‘Racial Equity in Algorithmic Criminal Justice’ (2019) 68 Duke LJ 1043; Sandra G Mayson, ‘Bias In, Bias Out’ (2019) 128 Yale LJ 2218. Some problematise automation deontologically. See eg Katrina Geddes, ‘The Death of the Legal Subject: How Predictive Algorithms Are (Re)constructing Legal Subjectivity’ (2023) 25 Vanderbilt Journal of Entertainment & Technology Law 1; Meg Leta Jones, ‘The Right to a Human in the Loop: Political Constructions of Computer Automation and Personhood’ (2017) 47 Social Studies of Science 216; Mireille Hildebrandt, ‘Privacy as Protection of the Incomputable Self: From Agnostic to Agonistic Machine Learning’ (2019) 20 Theoretical Inquiries in Law 83, 86.

7 Indeed, so does human decision making. Some scholars suggest, however, that in human decision making these challenges can be controlled for through administrative law. See eg Marion Oswald, ‘Algorithm-Assisted Decision-Making in the Public Sector: Framing the Issues Using Administrative Law Rules Governing Discretionary Power’ (2018) 376(2128) Philosophical Transactions of the Royal Society A 20170359; Frank Pasquale and Glyn Cashwell, ‘Prediction, Persuasion, and the Jurisprudence of Behaviourism’ (2018) 68 UTLJ 63, 66.

8 See also as part of this inquiry, Neli Frost, ‘The Global ‘Political Voice Deficit Matrix’’ (2023) 21(4) ICON 1041.

9 For examples of literature discussing how algorithmic decision-making undermines administrative legal principles, see, eg, in the US context, Danielle Keats Citron, ‘Technological Due Process’ (2008) 85 Washington University Law Review 1249; Cary Coglianese and David Lehr, ‘Transparency and Algorithmic Governance’ (2019) 71 Adm L Rev 1; David Freeman Engstrom and Daniel E Ho, ‘Algorithmic Accountability in the Administrative State’ (2020) 37 Yale J on Reg 800; Joshua A Kroll and others, ‘Accountable Algorithms’ (2017) 165 U Penn L Rev 633. See also *State of Wisconsin v Loomis* 881 NW2d 749 (Wis 2016), where the court found the COMPAS tool did not violate the defendant’s due process rights. In the UK and European contexts, see e.g., Jennifer Cobbe, ‘Administrative Law and the Machines of Government: Judicial Review of Automated Public-Sector Decision-Making’ (2019) 39 LS 636; Zalnieriute, Bennett Moses and Williams (n 2).

10 Etienne Wenger, *Communities of Practice: Learning, Meaning, and Identity* (CUP 1998).

11 I draw here mainly on the work of Philip Pettit. See Philip Pettit, *On the People’s Terms: A Republican Theory and Model of Democracy* (CUP 2012); Philip Pettit, *Republicanism: A Theory of Freedom and Government* (OUP 1999).

12 Martin Loughlin, *The Idea of Public Law* (OUP 2004).

13 Jürgen Habermas, *The Structural Transformation of the Public Sphere: An Inquiry into a Category of Bourgeois Society* (Thomas Burger tr, MIT Press 1991).

14 See eg the Kantian principle of ‘publicity’ introduced in *Perpetual Peace*, in the form of a ‘publicity test’: ‘All actions relating to the right of other human beings are wrong if their maxim is incompatible with publicity.’ Immanuel Kant, *Toward Perpetual Peace and Other Writings on Politics, Peace, and History* (Pauline Kleingeld ed and intro, David L Colclasure tr, Yale UP 2006) 104 (8: 381).

15 John Stuart Mill employed the concept—like Kant—to denote the value of transparency, but used it also in relation to public discourse and not only government action. John Stuart Mill, *Considerations on Representative Government* (Parker and Bourn 1861) 109–10. See also Hannah Arendt, *The Human Condition* (2nd edn, University of Chicago Press 1958); Habermas (n 13); Nancy Fraser, ‘Rethinking the Public Sphere: A Contribution to the Critique of Actually Existing Democracy’ (1990) 25 Social Text 56, 74–7; Iris Marion Young, *Inclusion and Democracy* (OUP 2000) 169–70.

16 Jeremy Waldron, ‘Can There Be a Democratic Jurisprudence?’ (2009) 58 Emory LJ 675; Benedict Kingsbury, ‘International Law as Inter-Public Law’, (2009) 49 Nomos 167, 178–9. See also Benedict Kingsbury and Nahuel Maisley, ‘Infrastructures and Laws: Publics and Publicness’ (2021) 17 Annual Review of Law and Social Science 353, 362.

17 For example, publicness in the Kantian formulation is a descriptor for desired features of political action by those in power, where the notion of ‘public’ is not the opposite of ‘private’, but rather the opposite of hidden or undisclosed: desired political action is open, communicable, transparent or knowable to the public. See Kevin R Davis, ‘Kantian “Publicity” and Political Justice’ (1991) 8 History of Philosophy Quarterly 409. See also Domingo García-Marzá and Castello de la Plana, ‘Kant’s Principle of Publicity: The Intrinsic Relationship Between the Two Formulations’ (2012) 103 Kant-Studien 96. In Hannah Arendt’s and Jürgen Habermas’s accounts of the ‘public realm’ and the ‘public sphere’, ‘publicity’ also captures the essence of politics as a mode of existence that takes place in the space between plural consociates through dialogical interactions, the presentation of diverse viewpoints, and reason-giving. See Arendt (n 15); Habermas (n 13). In the work of Nancy Fraser and Iris Marion Young, ‘publicity’ refers not only to the *process* of public discourse and accessibility thereof, but also to its *substance*: to its attention to matters that are of concern to everyone or that relate to a common good of a democratic community. See Fraser (n 15); Young (n 15). In the work of Jeremy Waldron and Benedict Kingsbury, ‘publicity’ was replaced by ‘publicness’, and evolved to denote an aspiration for, or quality of, *law* in a democracy. See Waldron (n 16); Kingsbury (n 16).

18 For a classic and potent critique, see Gerald E Frug, ‘The Ideology of the Bureaucracy in American Law’ (1984) 97 Harv L Rev 1276.

19 See Pettit, *On the People’s Terms* (n 11) and Pettit, *Republicanism* (n 11).

20 My theory of publicness also aligns with Chantal Mouffe’s agonistic theory of democracy. See Chantal Mouffe, *On the Political* (Routledge 2005). I focus, however, on neo-republicanism because it offers a more concrete vision of how agonistic spaces help constitute political communities as publics.

21 See a summary of Pettit’s theory in Pettit, *On the People’s Terms* (n 11) 293–310. See also Pettit, *Republicanism* (n 11) 55.

22 Bureaucracies are not exclusively a feature of the state but also of supranational organisations, as scholars of global governance would point out. See generally Benedict Kingsbury, Nico Krisch and Richard B Stewart, ‘The Emergence of Global Administrative Law’ (2005) 68 LCP 15.

23 Loughlin (n 12) ch 2.

24 ibid 156.

25 ibid 157, ch 3.

26 ibid ch 4. On ‘public agency’, see Timothy Endicott and Karen Yeung, ‘The Death of Law? Computationally Personalized Norms and the Rule of Law’ (2022) 72 UTLJ 373.

27 Loughlin (n 12) 156.

28 ibid 163.

29 Anthony Downs, ‘The Public Interest: Its Meaning in A Democracy’ (1962) 29 Social Research 1. For another canonical non-monolithic account of public interests see John Dewey, *The Public and Its Problems: An Essay in Political Inquiry* (Henry Holt 1927).

30 Downs (n 29); Loughlin (n 12) 157.

31 Loughlin (n 12) 152.

32 Wenger, *Communities of Practice* (n 10) 3.

33 Etienne Wenger, ‘Communities of Practice: Learning as a Social System’ (Systems Thinker) <https://thesystemsthinker.com/communities-of-practice-learning-as-a-social-system/> accessed 5 July 2024.

34 Wenger, *Communities of Practice* (n 10) 3.

35 On the use of this term in the context of public decision making, see Avihay Dorfman and Alon Harel, ‘The Case Against Privatization’ (2013) 41 Philosophy & Public Affairs 67.

36 Elizabeth Fisher and Sidney A Shapiro, *Administrative Competence: Reimagining Administrative Law* (CUP 2020). See also Bernardo Zacka, *When the State Meets the Street: Public Service and Moral Agency* (Harvard UP 2017).

37 Fisher and Shapiro (n 36) 52 (quoting Jerry Mashaw, emphasising that the experience of the system ‘resides in what one might call the feel or craft of decision makers’: Jerry L Mashaw, *Bureaucratic Justice: Managing Social Security Disability Claims* 67 (Yale UP 1983)). See also Zacka (n 36) 154 (describing street-level bureaucrats as ‘participants in a dense network of informal relationships’ that ‘play a central role in shaping the moral perception of individual bureaucrats and in setting the assumptions on the basis of which their moral reasoning takes place’); Simon Halliday, *Judicial Review and Compliance with Administrative Law* (Hart Publishing 2004) 41 (‘The decision-making process is better conceived as a network of bureaucrats exercising discretion, culminating in a final outcome which is highly contingent on the discretion of multiple actors’).

38 Fisher and Shapiro (n 36) 47.

39 ibid 50.

40 Michael Polanyi, *Personal Knowledge: Towards a Post-Critical Philosophy* (Routledge & Kegan Paul 1958).

41 Michael Oakeshott, *Rationalism in Politics and Other Essays* (Methuen & Co. 1962) 8–11.

42 Polanyi (n 40) 54–5.

43 ibid 50 (emphasis added).

44 See n 31 and accompanying text.

45 Zacka (n 36) 156.

46 Fisher and Shapiro (n 36) 52, quoting Mashaw at footnote 98 67.

47 ibid 54.

48 James C Scott, *Seeing Like a State: How Certain Schemes to Improve the Human Condition Have Failed* (Yale UP 2020) (the reference to Scott might seem perverse, given that Scott contrasts local knowledge with the ‘abstract knowledge deployed by the state and its technical agencies’, 311), but it is precisely this distinction that I want to dispel). See also Clifford Geertz, *Local Knowledge: Further Essays in Interpretive Anthropology* (3rd edn, Basic Books 1985).

49 Fisher and Shapiro (n 36) 53.

50 See Geertz (n 48) 153 (‘the community as the shop in which thoughts are constructed and deconstructed’).

51 Fisher and Shapiro (n 36) 55–8. See also Zoltan P Majdik and William M Keith, ‘Expertise as Argument: Authority, Democracy, and Problem-Solving’ (2011) 25 Argumentation 371, 374.

52 Sir Geoffrey Vickers, *The Art of Judgment: A Study of Policy Making* (Sage Publications 1995).

53 Fisher and Shapiro (n 36) 59 (quoting Thomas O McGarity writing about the EPA’s expertise ‘in making things happen’: Thomas O McGarity, ‘The Internal Structure of EPA Rulemaking’ (1991) 54 LCP 57, 62).

54 Dorfman and Harel (n 35) 82–3; Fisher and Shapiro (n 36) 90.

55 Jack M Balkin and Sanford Levinson, ‘The Canons of Constitutional Law’ (1998) 111 Harv L Rev 964, 980 (writing in a different context).

56 Jamal Greene, ‘The Anticanon’ (2011) 125 Harv L Rev 379, 381.

57 Gillian E Metzger and Kevin M Stack, ‘Internal Administrative Law’ (2017) 115 Mich L Rev 1239.

58 ibid 1239, 1248, 1254, 1265.

59 Dorfman and Harel (n 35) 83.

60 Fisher and Shapiro (n 36) 94.

61 This ethos is often spoken about in the context of ‘accountability’. See eg Fisher and Shapiro (n 36) 67, where administrative accountability is treated as ‘an axiomatic feature of a polity committed to democratic constitutionalism’. Indeed, accountability is closely related to the idea embodied in publicness, of decisions being made in the name of the public. On accountability as a contested term, see Patricia Day and Rudolph Klein, *Accountabilities, Five Public Services* (Tavistock Publications 1987). In their empirical research on how accountability is perceived in different agencies, Day and Klein conclude: ‘Lastly, many members tended to define accountability in terms of their responsibility, either to the community being served or to use their own sense of what was sensible or proper: they internalized accountability, as it were, as a general duty to pursue public good according to their own criteria of what was right … They saw themselves as trustees or tribunes rather than delegates. It is this perhaps which helps explain a pattern running right across our services: the emphasis among members on seeing accountability, answerability, or responsibility as being directed *to* the “community” at large’ (229).

62 From a political science perspective, the administrative law lens on political control of the bureaucracy has been limited in focusing on judicial review. See Mathew D McCubbins, Roger G Noll and Barry R Weingast, ‘Administrative Procedures as Instruments of Political Control’ (1987) 3 J L Econ & Organ 243, 245. In political science, two relevant traditions stand out: ‘overhead democracy’, concerned with ‘how to reconcile the necessary existence of an unelected, technocratic, and meritorious bureaucracy with the precepts of representative democracy and government by popular will’; and ‘classic public administration’, focused on the importance of expertise for determining and executing the public will. See Samuel Workman, *The Dynammics of Bureaucracy in the US Government: How Congress and Federal Agencies Process Information and Solve Problems* (CUP 2015) 20.

63 Katherine J Strandburg, ‘Rulemaking and Inscrutable Automated Decision Tools’ (2019) 119 Colum L Rev 1851.

64 Dorfman and Harel (n 35) 81.

65 ibid 84.

66 Loughlin (n 12).

67 John D Huber, Charles R Shipan and Madelaine Pfahler, ‘Legislatures and Statutory Control of Bureaucracy’ (2001) 45 American Journal of Political Science 330, 330 (‘Understanding this relationship is essential to democratic theory, as it focuses attention on the legitimacy of the role played by unelected policymakers in a representative democracy’). Such baked-in engagements are discussed by Fisher and Shapiro in relation to administrative accountability, but this term fails to fully capture the principal idea underpinning the reification of public interests—the idea that public decisions are made *in the name of* the public or the polity. Fisher and Shapiro (n 36) 67.

68 Workman (n 62) ch 2.

69 See eg the functions performed by the Government Accountability Office: Jesse M Cross and Abbe R Gluck, ‘The Congressional Bureaucracy' (2020) 168 U Penn L Rev 1541, 1587–94.

70 See eg Jason A Macdonald, ‘Limitation Riders and Congressional Influence over Bureaucratic Policy Decisions’ (2010) 104 American Political Science Review 766, 766 (on ‘limitation riders’).

71 McCubbins, Noll and Weingast (n 62) 255.

72 ibid 255; Metzger and Stack (n 57) 1266.

73 The Administrative Procedure Act, 5 USC §§ 551–559.

74 Martino Maggetti and Yannis Papadopoulos, ‘The Principle–Agent Framework and Independent Regulatory Agencies’ (2018) 16 Political Studies Review 172.

75 McCubbins, Noll and Weingast (n 62) 258.

76 See the Federal Advisory Committee Act, Pub L 92-463, 6 October 1972, 86 Stat 770.

77 Steven J Balla and John R Wright, ‘Interest Groups, Advisory Committees, and Congressional Control of the Bureaucracy’ (2001) 45 American Journal of Political Science 799, 800.

78 Huber, Shipan and Pfahler (n 67) 332.

79 Lanny W Martin and Georg Vanberg, ‘Coalition Policymaking and Legislative Review’ (2005) 99 American Political Science Review 93, 97 (mechanisms constraining the capacity of ministers to ‘move policy in directions favored by their respective parties’). See also Fabio Franchino and Bjørn Høyland, ‘Legislative Involvement in Parliamentary Systems: Opportunities, Conflict, and Institutional Constraints’ (2009) 103 American Political Science Review 607; John D Huber and Charles R Shipan, *Deliberate Discretion?**The Institutional Foundations of Bureaucratic Autonomy* (CUP 2002) (a comparative theory of delegation explaining the factors driving politician’ choices between detailed and vague statutes).

80 Workman (n 62) 31.

81 Cyril Benoît, ‘Legislatures and the Administrative State: Political Control, Bureaucratic Politics and Public Accountability’ in Cyril Benoît and Olivier Rozenberg (eds), *Handbook of Parliamentary Studies: Interdisciplinary Approaches to Legislatures* (Edward Elgar Publishing 2020) 268; Maggetti and Papadopoulos (n 74) 178.

82 Workman (n 62) 32.

83 For a similar claim, specific to the US context (and in relation also to politically appointed heads of administrative agencies), see Jon D Michaels, ‘An Enduring, Evolving Separation of Powers’ (2015) 115 Colum L Rev 515, 543 (‘the independent and much relied-upon civil service has institutional, cultural, and legal incentives to insist that agency leaders follow the law, embrace prevailing scientific understandings, and refrain from partisan excesses. That is to say, these professional civil servants regularly do have reason to "choose" to hold agency leaders accountable.'). I disagree, however, with Michaels’s claim that ‘if the civil service … reigned supreme, administration action would be an arid technocratic endeavor largely insulated from presidential mandates and public concerns’ (554).

84 Benoît (n 81) 258. See my comment on publicness and accountability (n 61).

85 See Frug (n 18). See also David Kennedy, *A World of Struggle: How Power, Law, and Expertise Shape Global Political Economy* (Princeton UP 2016).

86 See eg Oakeshott’s preoccupation with rationalist politics and their drying up of the mind, Oakeshott (n 41) 31; Polanyi’s critique of the obsession with objectivist epistemology, Polanyi (n 40); and Vickers’s worry about technical rationality, Vickers (n 52).

87 David Lehr and Paul Ohm, ‘Playing with the Data: What Legal Scholars Should Learn About Machine Learning’ (2017) 51 UC Davis L Rev 653, 669.

88 Others use the terms ‘data-driven regulation’: Mireille Hildebrandt, ‘Algorithmic Regulation and the Rule of Law’ (2018) 376(2128) Philosophical Transactions of the Royal Society A 20170355; or ‘algorithmic regulation’: Karen Yeung, ‘Algorithmic Regulation: A Critical Interrogation’ (2018) 12 Regulation & Governance 505, 507; Karen Yeung and Martin Lodge, ‘Algorithmic Regulation, An Introduction’ in Karen Yeung and Martin Lodge (eds), *Algorithmic Regulation* (OUP 2019) 5.

89 Yeung, ‘Algorithmic Regulation’ (n 88) 506.

90 Julia Angwin, ‘Sample-COMPAS-Risk-Assessment-COMPAS-“CORE”’ <www.documentcloud.org/documents/2702103-Sample-Risk-Assessment-COMPAS-CORE.html#document/p1/a296558> accessed 5 July 2024.

91 Clinical evaluations are based on ‘decision-makers’ subjective judgments, observations, and experiences’: Alyssa M Carlson, ‘The Need for Transparency in the Age of Predictive Sentencing Algorithms’ (2017) 103 Iowa L Rev 303, 305. The extent to which computational models really only ‘assist’ in decision making is compounded by the phenomenon of automation bias, whereby decision makers tend to comply with algorithms as these are viewed as ‘outperforming human expertise’: Hildebrandt, ‘Algorithmic Regulation’ (n 88) 2.

92 See eg Johana Bhuiyan, ‘LAPD Ended Predictive Policing Programs amid Public Outcry. A New Effort Shares Many of Their Flaws’ *The Guardian* (8 November 2021) <www.theguardian.com/us-news/2021/nov/07/lapd-predictive-policing-surveillance-reform> accessed 5 July 2024; Matt Burgess, ‘UK Police Are Using AI to Inform Custodial Decisions—but It Could Be Discriminating against the Poor’ (WIRED 1 March 2018) <www.wired.co.uk/article/police-ai-uk-durham-hart-checkpoint-algorithm-edit> accessed 5 July 2024; Andrew Guthrie Ferguson, ‘Policing Predictive Policing’ (2017) 94 Wash UL Rev 1113.

93 See eg Janna Brancolini, ‘Italy Turns to AI to Find Taxes in Cash-First, Evasive Culture’ (Bloomberg 31 October 2022) <https://news.bloombergtax.com/daily-tax-report-international/italy-turns-to-ai-to-find-taxes-in-cash-first-evasive-culture> accessed 5 July 2024.

94 See eg Jennifer Raso, ‘Displacement as Regulation: New Regulatory Technologies and Front-Line Decision-Making in Ontario Works’ (2017) 32 Canadian Journal of Law & Society 75; Robert N Charette, ‘Michigan’s MiDAS Unemployment System: Algorithm Alchemy Created Lead, Not Gold’ (*IEEE Spectrum* 24 January 2018) <https://spectrum.ieee.org/michigans-midas-unemployment-system-algorithm-alchemy-that-created-lead-not-gold> accessed 5 July 2024.

95 See eg David Jackson and Gary Marx, ‘Data Mining Program Designed to Predict Child Abuse Proves Unreliable, DCFS Says’ *Chicago Tribune* (6 December 2017) <www.chicagotribune.com/investigations/ct-dcfs-eckerd-met-20171206-story.html> accessed 5 July 2024; Virgina Eubanks, ‘A Child Abuse Prediction Model Fails Poor Families’ (WIRED,15 January 2018) <www.wired.com/story/excerpt-from-automating-inequality> accessed 5 July 2024; Niamh McIntyre and David Pegg, ‘Councils Use 377,000 People’s Data in Efforts to Predict Child Abuse’ *The Guardian* (16 September 2018) <www.theguardian.com/society/2018/sep/16/councils-use-377000-peoples-data-in-efforts-to-predict-child-abuse> accessed 5 July 2024; Dan Hurley, ‘Can an Algorithm Tell When Kids Are in Danger?’ *New York Times* (2 January 2018) <www.nytimes.com/2018/01/02/magazine/can-an-algorithm-tell-when-kids-are-in-danger.html> accessed 5 July 2024; Alexandra Chouldechova and others, ‘A Case Study of Algorithm-Assisted Decision Making in Child Maltreatment Hotline Screening Decisions’ (2018) 81 Proceedings of Machine Learning Research 1 <http://proceedings.mlr.press/v81/chouldechova18a/chouldechova18a.pdf> accessed 5 July 2024.

96 For an overview of various use cases, see Coglianese and Ben-Dor (n 1). See also David Freeman Engstrom and others, ‘Government by Algorithm: Artificial Intelligence in Federal Administrative Agencies’, Report Submitted to the Administrative Conference of the United States (February 2020). Geddes offers a typology of the examples mentioned herein as pertaining to one of three distinct categories: (i) the automation of legal rules; (ii) the automation of legal standards; and (iii) prediction that informs the application of legal standards. Geddes (n 5) 17–21.

97 Yeung, ‘Algorithmic Regulation’ (n 88) 506.

98 ibid.

99 Jenna Burrell, ‘How the Machine “Thinks”: Understanding Opacity in Machine Learning Algorithms’ (2016) 3 Big Data & Society 1, 6. Or, in other words: the optimal ‘predictive relationship between inputs and outputs’. Yeung, ‘Algorithmic Regulation’ (n 88) 506. Lehr and Ohm offer a definition of machine learning as ‘an automated process of discovering correlations (sometimes alternatively referred to as relationships or patterns) between variables in a dataset, often to make predictions or estimates of some outcome’: Lehr and Ohm (n 87) 671 (unpacking machine learning into two workflows they term ‘playing with the data’ and ‘the running model’).

100 Brent Daniel Mittelstadt and others, ‘The Ethics of Algorithms: Mapping the Debate’ (2016) 3 Big Data & Society 1, 3 (‘Critically, the human operator does not need to understand the rationale of decision-making rules produced by the algorithm’). It is important to mention, however, that while machine learning systems can indeed autonomously optimise their objective function, this function—the model’s overall goals—still needs to be manually defined. See Brian Sheppard, ‘Warming Up to Inscrutability: How Technology Could Challenge Our Concept of Law’ (2018) 68 UTLJ 36, 44. See also Karen Yeung, ‘Why Worry about Decision-Making by Machine?’ in Yeung and Lodge, *Algorithmic Regulation* (n 88) 21, 22, qualifying these technologies as having ‘decisional autonomy’, ‘insofar as they are designed to adapt dynamically to their environment, hence the processing of inputs and the determination of outputs partly independent of their human designers’.

101
www.fatml.org/.

102 See eg Cathy O’Neill, *Weapons of Math Destruction: How Big Data Increases Inequality and Threatens Democracy* (Penguin Books 2017); Cynthia Dwork and others, ‘Fairness through Awareness’ ITCS ’12: Proceedings of the 3rd Innovations in Theoretical Computer Science Conference (2012) 214; Alexandra Chouldechova, ‘Fairness Prediction with Disparate Impact: A Study of Bias in Recidivism Prediction Instruments’ (2017) 5 Big Data 153; Burrell (n 99); Richard Berk and others, ‘Fairness in Criminal Justice Risk Assessments: The State of the Art’ (2021) 50 Sociological Methods & Research 3; Teresa Scantamburlo, Andrew Charlsworth and Nello Cristianini, ‘Machine Decisions and Human Consequences’ in Yeung and Lodge, *Algorithmic Regulation* (n 88) 49, 76.

103 See eg Daniel Greene, Anna Lauren Hoffmann and Luke Stark, ‘Better, Nicer, Clearer, Fairer: A Critical Assessment of the Movement for Ethical Artificial Intelligence and Machine Learning’ Proceedings of the 52nd Hawaii International Conference on System Sciences (2019); Sam Corbett-Davies and others, ‘The Measure and Mismeasure of Fairness’, arXiv: 1808.0023v3 [cs.CY] (14 August 2023). Others have offered socio-technical analyses leading to further solutions to the problem of unfairness. See eg Mateusz Dolata, Stefan Feuerriegel and Gerhard Schwabe, ‘A Sociotechnical View of Algorithmic Fairness’ (2022) 32 Information Systems Journal 754.

104 These conceptualisations are aligned with how European regulations of novel technologies frame the stakes in a ‘rights’ vocabulary in eg the General Protection Data Regulation (GDPR) or the AI Act, Regulation (EU) 2024/1689 of the European Parliament and of the Council of 13 June 2024 laying down harmonised rules on artificial intelligence and amending Regulations (EC) No 300/2008, (EU) No 167/2013, (EU) No 168/2013, (EU) 2018/858, (EU) 2018/1139 and (EU) 2019/2144 AND Directives 2014/90/EU, (EU) 2016/797 and (EU) 2020/1828.

105 Elettra Bietti, ‘From Ethics Washing to Ethics Bashing: A View on Tech Ethics from Within Moral Philosophy’ (FAT* January 2020) 210 (‘ethics washing’ means ‘the instrumentalization of ethical language by tech companies’).

106 See eg scholarship on the GDPR right relating to automation: Bryce Goodman and Seth Flaxman, ‘European Union Regulations on Algorithmic Decision-Making and a “Right to Explanation”’ arXiv: 1606.08813v3 [stat.ML] (31 August 2016), https://arxiv.org/pdf/1606.08813.pdf. For scholarship on the *right* to non-discrimination, see eg Solon Barocas and Andrew D Selbst, ‘Big Data’s Disparate Impact’ (2016) 104 Cal L Rev 671; Natalia Criado and Jose M Such, ‘Digital Discrimination’ in Yeung and Lodge, *Algorithmic Regulation* (n 88) 82; Jeremias Adams-Prassl, Reuben Binns and Aislinn Kelly-Lyth, ‘Directly Discriminatory Algorithms’ (2023) 86 MLR 144.

107 See eg Karen Yeung, Andrew Howes and Ganna Pogrebna, ‘AI Governance by Human Rights-Centered Design, Deliberation, and Oversight: An End to Ethics Washing’ in Markus D Dubber, Frank Pasquale, and Sunit Das (eds), *The Oxford Handbook of Ethics of AI* (OUP 2020) 76; Mark Latonero, ‘Governing Artificial Intelligence: Upholding Human Rights & Dignity’ (Data & Society 2018) <https://datasociety.net/wp- content/uploads/2018/10/DataSociety_Governing_Artificial_Intelligence_Upholding_Human_Rights.pdf> accessed 5 July 2024; Lorna McGregor, Daragh Murray, and Vivian Ng, ‘International Human Rights Law as a Framework for Algorithmic Accountability’ (2019) 68 ICLQ 309; Access Now, ‘Human Rights in the Age of Artificial Intelligence’ <www.accessnow.org/wp-content/uploads/2018/11/AI-and-Human-Rights.pdf> accessed 5 July 2024; Kate Saslow and Philippe Lorenz, ‘Artificial Intelligence Needs Human Rights: How the Focus on Ethical AI Fails to Address Privacy, Discrimination and Other Concerns’ (30 September 2019); Article 19, ‘Governance with Teeth: How Human Rights Can Strengthen FAT and Ethics Initiatives on Artificial Intelligence’ <www.article19.org/wp-content/uploads/2019/04/Governance-with-teeth_A19_April_2019.pdf> accessed 5 July 2024.

108 See n 9 and accompanying text.

109 See n 6 and accompanying text.

110 Salomé Viljoen, ‘A Relational Theory of Data Governance’ (2021) 131 Yale LJ 573.

111 See eg the emphasis on fundamental rights in the EU’s AI Act (n 104).

112 Adrian Mackenzie, ‘The Production of Prediction: What Does Machine Learning Want?’ (2015) 18 European Journal of Cultural Studies 429, 432.

113 Endicott and Yeung (n 26).

114 Mackenzie (n 112) 433.

115 Charles E Lindblom, ‘The Science of “Muddling Through”’ (1959) 19 PAR 79.

116 Loughlin (n 12).

117 Vickers (n 52) 43, 48.

118 See n 23 and accompanying text.

119 Vickers (n 52).

120 Zacka (n 36) 157.

121 Fisher and Shapiro (n 36) 54 (quoting Giandomenico Majone in referring to the skills of practice reasoning as ‘not algorithmical but argumentative’, involving ‘the ability to probe assumptions critically, to produce and evaluate evidence, to keep many threads in hand, to draw for an argument many disparate sources, to communicate effectively’: Giandomenico Majone, *Evidence, Argument and Persuasion in the Policy Process* (Yale UP 1989) 21–2.

122 Loughlin (n 12) 156.

123 Amnesty International (n 3) 5.

124 ibid.

125 ibid 15.

126 ibid

127 ibid 16.

128 ibid 6.

129 Making a similar point, see Vickers (n 52) 86 (‘The value judgments of men and societies cannot be *proved* correct or incorrect; they can only be *approved* as right or *condemned* as wrong by the exercise of another value judgment’). Dispelling the fact/value distinction, see also Geertz (n 48).

130 Day and Klein (n 61) 59–63. See text to nn 27–31.Emphasis in original.

131 See n 86 and accompanying text.

132 Raso (n 94) 79 (how novel regulatory technologies deskill frontline administrators and how these workers ‘reassert themselves as skilled decision-makers by tweaking data inputs to soften SAMS’s prescriptive decision-making approach’).

133 Pettit, *On the People’s Terms* (n 11) 270 (writing in a different context).

134 Strandburg (n 63) 1861 (‘Unfortunately, reliance on found data leaves rulemakers at the mercy of whatever feature sets and outcome variables happen to have been collected’).

135 Vickers (n 52).

136 Strandburg (n 63) 1862.

137 ibid 1863.

138 Ignas Kalpokas, *Algorithmic Governance: Politics and Law in the Post-Human Era* (Palgrave Macmillan 2019) 71.

139 Cade Metz, ‘Can a Machine Learn Morality?’ *New York Times* (19 November 2021) <www.nytimes.com/2021/11/19/technology/can-a-machine-learn-morality.html> accessed 5 July 2024.

140 Eubanks (n 95).

141 Sally Ho and Garance Burke, ‘An Algorithm that Screens for Child Neglect Raises Concerns’ (AP 29 April 2022) <https://apnews.com/article/child-welfare-algorithm-investigation-9497ee937e0053ad4144a86c68241ef1> accessed 5 July 2024.

142 ibid.

143 ibid. See also Eubanks (n 95).

144 Ho and Burke (n 141).

145 Yeung, ‘Algorithmic Regulation’ (n 88) 509 (quoting H Ekbia and others, ‘Big Data. Bigger Dilemmas: A Critical Review’ (2015) 68 JASIST 1523).

146 Ho and Burke (n 141) (quoting Brian Chor, researcher at the University of Chicago’s Chaplin Hall).

147 Dorfman and Harel (n 35).

148 Strandburg (n 63) 1872.

149 Margaret Jane Radin, *Contested Commodities: The Trouble with Trade in Sex, Children, Body Parts and Other Things* (Harvard UP 1996) 14.

150 See Loughlin (n 12).

151 Benoît (n 81) 261–2 (arguing most research on the legislator–administrator relationship has been US-focused). For an in-depth analysis of the need for comparative administrative law, see Oren Tamir, ‘Our Parochial Administrative Law’ (2024) 97 S Cal L Rev 101.

152 B Guy Peters, ‘Introduction’ in Aaron Wildavsky, *The Art and Craft of Policy Analysis* (Reissued with a new introduction by B Guy Peters) (Springer 2017) xvii, xix (‘it is important to remember that public policy and policy analysis are inherently political’).

153 See eg Paul R Vekuil, *Outsourcing Sovereignty: Why Privatization of Government Functions Threatens Democracy and What Can We Do About It* (CUP 2007).

154 On accountability in automated decision making, see eg Engstrom and Ho (n 9); Kroll and others (n 9).

155 Pettit, *On the People’s Terms* (n 11) (mechanisms for democratic influence and control).

156 Other legal instruments prominently enlisted to mitigate the challenges of algorithmic decision making include the GDPR, the Equality Act 2010 and the Human Rights Act 1998 in the UK (see Paul Craig, *Administrative Law* (9th edn, Sweet & Maxwell 2021) 421–2) and the due process clause of the Fourteenth Amendment in the United States (see Geddes (n 5) 61–3).

157 Craig (n 156). In the US context, see Geddes (n 5) 58–67. For how administrative law could be adjusted to address algorithmic decision making, see Rebecca Williams, ‘Rethinking Administrative Law for Algorithmic Decision Making’ (2022) 42 OJLS 468.

158 Craig (n 156).

159 eg US doctrines of ‘non-delegation’ aiming to ‘preserve the distribution of power between the different branches of government’ come closer to this ideal, albeit still imperfectly: Geddes (n 5) 59.

